# Genome sequence of *Gilbertella persicaria* strain SHK1000 isolated from fig fruit

**DOI:** 10.1128/mra.00467-25

**Published:** 2026-03-06

**Authors:** Masahito Nakano

**Affiliations:** 1 Toyo Institute of Food Technology, Hyogo, Japan; University of California Riverside, Riverside, California, USA

**Keywords:** *Gilbertella persicaria*, *Ficus carica*, soft rot

## Abstract

*Gilbertella persicaria* is a phytopathogenic fungus that causes soft rot in several fruit crops. Here, I report the draft genome sequence of *G. persicaria* strain SHK1000 isolated from fig fruit, and consisting of total length 30.9 Mbp across 60 contigs with N_50_ 3.6 Mbp and 99.2% BUSCO completeness.

## ANNOUNCEMENT

*Gilbertella persicaria* is a phytopathogenic fungus that causes soft rot in economically important fruit crops, such as peaches, strawberries, and Japanese pears (1–3). This fungus produces endoglucanases and exoglucanases that compromise the structural integrity of the cell wall, leading to severe soft rot in host plants (4). In a previous study, the fungal strain SHK1000 was isolated at the Toyo Institute of Food Technology experimental orchard (34°49′08.60″N, 135°24′11.60″E) in Hyogo Prefecture, Japan, from fig (*Ficus carica* L.) fruit exhibiting soft rot symptoms. The strain was taxonomically identified as *G. persicaria* based on the multilocus analysis of the internal transcribed spacer of rDNA, 28S rDNA large subunit, and actin (LC859118–LC859123) (5). The strain was deposited at the Research Center of Genetic Resources, National Agriculture and Food Research Organization (Ibaraki, Japan) with accession number MAFF248114. Here, I report the draft genome sequence of *G. persicaria* strain SHK1000, which will facilitate further investigations of the pathogenicity of the fungus on fig.

The strain was grown on potato dextrose agar (PDA) every six months and maintained at 4°C. For genome sequencing, the strain was cultured on PDA for three days and then in 100 mL of liquid potato dextrose broth for two days at 25°C on a rotary shaker at 150 rpm. Cultured cells were collected by gravity filtration using a filter paper No. 2 (Advantec, Japan), and genomic DNA was extracted using the NucleoBond HMW DNA kit (Takara Bio, Japan) and purified using DNA Clean Beads (MGI Tech, China). An ultralow DNA input library was prepared using the SMRTbell gDNA Sample Amplification kit (Pacific Biosciences, USA) in combination with the SMRTbell Prep kit 3.0 (Pacific Biosciences). In parallel, a PCR-free DNA library was prepared using the SMRTbell Prep kit 3.0, with genomic DNA fragmented to 10–25 Kbp using Megaruptor 3 (Diagenode, Belgium). These libraries were subsequently sequenced on a Revio (Pacific Biosciences) by the Bioengineering Lab. Co., Ltd. (Kanagawa, Japan). SMRT Link v.13.1.0.221970 was used to remove overhang adapter sequences from the reads and obtain high-fidelity reads with average quality values > Q20, yielding 296,782 HiFi reads with N_50_ 10,166 bp. The default parameters were used for all software except otherwise noted. The HiFi reads were filtered to a length >5,000 bp using SeqKit v.2.10.0 (6) and subsequently assembled *de novo* using Hifiasm v.0.21.0-r686 (7). QUAST-LG v.5.3.0 (8) was used to verify the quality of the assembled genome sequences. Genome completeness was estimated using Benchmarking Universal Single-Copy Orthologs (BUSCO) v.5.7.0 with the mucorales_odb10 data set (9). The average nucleotide identity (ANI) was calculated between *G. persicaria* SHK1000 and other strains using FastANI v.1.34 (10), resulting in ANI values of 99.6% for the *G. persicaria* CBS 190.32 strain (GCA_025201335.1) and 99.1% for the TFLB-J strain (GCA_024294565.1). A summary of the genome assembly data is presented in Table 1.

**TABLE 1:** Summary of the *G. persicaria* genome

Genome feature	SHK1000
Total length (bp)	30,922,490
Number of contigs	60
Coverage (×)	65
N_50_ (bp)	3,665,012
L_50_	4
GC content (%)	37.28
BUSCOs (% out of 2,449 genes in mucorales_odb10)	
Complete and single-copy BUSCOs	99.0
Complete and duplicated BUSCOs	0.2
Fragmented BUSCOs	0.1
Missing BUSCOs	0.7

## Data Availability

The *G. persicaria* SHK1000 genome sequence was deposited in DDBJ/EMBL/GenBank under accession number PRJDB20706, and the raw sequencing reads were submitted to the DDBJ Sequence Read Archive (DRA) under accession numbers DRR681652 (PCR-free DNA library) and DRR668257 (ultralow DNA input library). The assembled genome sequences are available in DDBJ under accession number BAAHPX000000000.
